# Oridonin inhibited epithelial-mesenchymal transition of laryngeal carcinoma by positively regulating LKB1/AMPK signaling

**DOI:** 10.7150/ijms.92182

**Published:** 2024-01-21

**Authors:** Bo Kou, Yuhan Shi, Zhaoyue Zhou, Yanning Yun, Qun Wu, Jinsong Zhou, Wei Liu

**Affiliations:** 1 Department of Otorhinolaryngology-Head&Neck Surgery, The First Affiliated Hospital of Xi'an Jiaotong University, Xi'an, Shaanxi 710061, China.; 2 Department of Legal Affairs, Shaanxi Provincial People's Hospital, Xi 'an 710054, China.; 3 Department of Human Anatomy, Histology and Embryology, School of Basic Medical Sciences, Xi'an Jiaotong University Health Science Center, Xi'an, Shaanxi 710061, China.; 4 Key Laboratory of Environment and Genes Related to Diseases, Xi'an Jiaotong University, Ministry of Education of China, Xi'an, Shaanxi 710061, China.; 5 Department of Vascular Surgery, The First Affiliated Hospital of Xi'an Jiaotong University, Xi'an, Shaanxi 710061, China.

**Keywords:** Oridonin, Laryngeal carcinoma, EMT, LKB1, AMPK

## Abstract

Oridonin is the main bioactive component of Rabdosia rubescens, and its anticancer activity has been reported in a variety of cancers. However, the molecular mechanism of oridonin in laryngeal carcinoma remains unclear. In the present study, the cytotoxic effect of oridonin on laryngeal carcinoma Hep-2 and TU212 cell lines were initially detected by modified MTT assay. The results showed that oridonin had a dose-dependent anti-proliferative effect on laryngeal carcinoma Hep-2 and TU212 cells. Next, we found that oridonin significantly inhibited the migration and invasion of human laryngeal carcinoma Hep-2 and TU212 cell lines by wound healing assay and transwell assay. Subsequently, the results of quantitative real-time PCR assay and western blotting assay confirmed that oridonin upregulated the expression of E-cadherin while downregulated the expression of N-cadherin in a concentration-dependent manner at mRNA and protein levels. In addition, phosphorylation levels of liver kinase B1 (p-LKB1) and AMP-activated protein kinase (p-AMPK) were also elevated upon oridonin treatment. To further verify the role of LKB1/AMPK signaling pathway in laryngeal carcinoma, overexpression of LKB1 was constructed by plasmid transfection. The data exhibited that overexpression of LKB1 could further reinforce the increase of E-cadherin level and decrease of N-cadherin level mediated by oridonin. Additionally, AMPK inhibitor compound C could reverse anti-metastatic effect of oridonin on laryngeal carcinoma, and antagonise EMT expression. In contrast, AMPK activator AICAR presented the opposite effect. In conclusion, our study revealed that oridonin could remarkably reverse the epithelial-mesenchymal transition of laryngeal carcinoma by positively regulating LKB1/AMPK signaling pathway, which suggested that oridonin may be a potential candidate for the treatment of laryngeal carcinoma in the future.

## Introduction

Laryngeal carcinoma (LC) is the most common malignant tumor of the throat, accounting for 1% to 5% of human tumors 1. Previous studies have shown that the pathogenic factors of laryngeal cancer include smoking, drinking, environmental pollution, viral infection, radiation, sex hormone metabolism disorders, gene mutations and so on 2. In recent years, due to the change of living environment and lifestyle, the incidence of laryngeal cancer has gradually increased in China 3. In the early stage, most patients with laryngeal cancer have no obvious symptoms, while in the middle and late stage, hoarseness, sputum and blood coughing, foreign bodies in the larynx, and lymph node enlargement may gradually appear. At present, the clinical treatment of laryngeal cancer is mainly based on surgery, chemo-radiotherapy, molecular targeting and other therapeutic methods. Although the condition of patients can be improved to a certain extent, the recurrence, metastasis and low survival rate of laryngeal cancer still affect the treatment of patients 4. The prognosis of patients with advanced laryngeal carcinoma remains not optimistic, and the 5-year survival rate is only 51% 5. Therefore, it is an urgent necessity to study the molecular mechanism of laryngeal carcinoma to explore effective therapeutic strategies.

Oridonin, a naturally occurring terpenoid, was mainly found in Rabdosia rubescens. In recent years, numerous studies have demonstrated that oridonin has a vital role in anti-inflammatory 6, anti-cancer 7, 8, antibacterial 9, anti-sepsis 10, neuroprotection 11, immune regulation and other pharmacological and physiological aspects 12. Among them, its anticancer activity has been widely reported in nasopharyngeal cancer 13, esophageal cancer 14, gastric cancer 15, colorectal cancer 16, 17, pancreatic cancer 18, gallbladder cancer 19, breast cancer 20, ovarian cancer and other malignant cancers 21, 22. And the anticancer mechanism of oridonin is involved with inhibition of proliferation, induction of apoptosis, autophagy and inhibition of migration and invasion 23. Previous study reported that oridonin induced G2/M phase arrest and apoptosis of human laryngeal carcinoma cells 24. And inhibition of EGFR could strengthen the apoptotic induction of oridonin in laryngeal carcinoma cells by activating oxidative stress 25. In our previous study, it's reported that oridonin could facilitate endoplasmic reticulum stress (ER stress) to induce laryngeal carcinoma cell apoptosis 26. Furthermore, cetuximab, an anti-EGFR monoclonal antibody, was found to reinforce oridonin-induced apoptosis by ER stress and mitochondrial pathway in laryngeal cancer 27-28. Nevertheless, the effect of oridonin on laryngeal carcinoma metastasis has not been clearly elucidated 21.

Human liver kinase (LKBI), mutated in Peutz-Jeghers syndrome (PJS), acts as a tumor suppressor in a variety of cancers 29. LKB1 encodes serine-threonine kinase (STK) 11, activates AMPK-activated protein kinase (AMPK) and its 13 superfamily members, and is involved in regulating a variety of biological processes such as cell polarity 30, cell cycle arrest 31, 32, embryonic development 33, apoptosis and bioenergy metabolism 34. Recent studies reported that LKB1/AMPK pathway participates in malignant cell transformation and tumor progression, and is involved in various cancer types including gastric cancer 35, non-small cell lung cancer 36, breast cancer 37, pancreatic cancer 38, and liver cancer 39, 40. The findings of our previous study uncovered that LKB1/AMPK signaling could suppress epithelial- mesenchymal transition of renal cell carcinoma upon thymoquinone treatment 41. However, whether LKB1/AMPK signaling pathway was correlated with the anticancer activity of oridonin in laryngeal carcinoma remains unclear. Herein, in the present study, we aimed to investigate the correlation between oridonin and laryngeal cancer metastasis, and its underlying molecular mechanism.

## Materials and methods

### Materials, reagents, and antibodies

Oridonin (C_20_H_28_O_6_) and dimethyl sulfoxide (DMSO) were obtained from Sigma-Aldrich (St. Louis, MO, USA). Primary rabbit monoclonal antibodies (diluted at 1:1,000) against phosphorylated-LKB1, phosphorylated- AMPK, E-cadherin (E-Ca), N-cadherin(N-Ca) and β-actin were purchased from Cell Signaling Technology, Inc (Beverly, MA, USA). AICAR and Compound C, OE-LKB1

### Cell lines and cell culture

Human laryngeal carcinoma Hep-2 and TU212 cell lines were purchased from American Type Culture Collection (Manassas, VA, USA). These two cell lines were cultured in RPMI 1640 medium and supplemented with 10% fetal bovine serum (FBS; Gibco, Grand Island, NY, USA), 100 µg/ml streptomycin and 100 U/ml penicillin (Invitrogen, Carlsbad, CA, USA). All cells were seeded under saturated humidity with 5% CO2, and 37°C.

### MTT assay

The modified MTT assay was used to explore the cell proliferation viability. Briefly, 1.0×10^4^ Hep-2 and TU212 cell lines were plated into 96-well plates at 200 μL per well, respectively. Upon the treatment with dimethyl sulfoxide (DMSO) or the increasing concentrations of oridonin for 24 h, 20 µl of MTT dye solution (5.0 mg ml^-1^) was added to each well with 180 µl medium for 4 h. Then cells were lysed with DMSO to dissolve the violet blue crystals. Then the optical density (OD) of each cell was measured at 490 nm wavelength on microplate reader (Bio-Rad, Hercules, CA, USA). The growth inhibitory rate was calculated as: [(OD_490control cells_ - OD_490treated cells_)/OD_490control cells_] ×100.

### Wound healing assay

Hep-2 and TU212 cells were plated onto six-well plates. The wounds were scratched across the monolayer with the tip of a 200-µl pipette when cell density reached 90 % confluence. After incubation in a serum-free medium with oridonin treatment, five different fields (100×) were randomly chosen from each scratch wound and visualized by microscope to assess the migratory capacity. Five independent experiments were performed.

### Transwell migration assay

Transwell migration assays was conducted upon oridonin treatment using Hep-2 and TU212 cells. Briefly, 6×10^4^ cells with 200 μl serum-free medium were added into the upper chamber (Millipore, Billerica, MA, USA), while 800 μl of 10% FBS-supplemented medium were subsequently plated onto the lower chamber. After incubation at 37 °C for 24 h, the cells that migrated to the lower surface of the filter were fixed in 4 % paraformaldehyde and stained with 0.1% crystal violet (Beyotime, Shanghai, China) for 10 min. Cells were counted in five random fields and visualized using an optical microscope at 100×magnification. Five independent experiments were performed.

### Matrigel invasion assay

The effect of oridonin on the invasion of Hep-2 and TU212 cells was detected by matrigel invasion assay with a Millicell chamber (Millipore, Billerica, MA, USA). The membrane with 8 μm pore size in the upper chamber was seeded with 50 μL of mixture (Matrigel: serum-free medium 1:5) with the rest of the steps being similar to the Transwell migration assay. Five independent experiments were performed.

### Quantitative real-time PCR assay

Total RNA was extracted from treated cells using TRIzol reagent (Invitrogen, Carlsbad, CA, USA) and reversely transcribed into complementary DNA (cDNA) using the PrimerScript RT reagent Kit (Takara, Dalian, China). Subsequently, the quantitative real-time PCR assay (qRT-PCR) was carried out by SYBR Green Master Mix. The primer sequences were as follows:

E-Cadherin (forward: 5'-CGAGAGCTACACGTTCACGG-3'; reverse: 5'-GGGTGTCGAGGGAAAAATAGG-3');

N-Cadherin (forward: 5'-TCAGGCGTCTGTAGAGGCTT-3'; reverse: 5'-ATGCACATCCTTCGATAAGACTG-3');

β-actin (forward: 5'-CATGTACGTTGCTATCCAGGC-3'; reverse: 5'-CTCCTTAATGTCACGCACGAT-3');

The n-fold change in mRNA expression was calculated according to the 2-^∆∆Ct^ method. Five independent experiments were performed.

### Western blotting

Briefly, Hep-2 and TU212 were collected upon certain treatment, and the proteins were extracted using protein lysis buffer. After centrifugation and denaturation, the extracts were subjected to Sodium Dodecyl Sulfate-polyacrylamide gel electrophoresis (10 % or 15 %) and transferred to polyvinylidene fluoride (PVDF) membranes (Millipore, Bedford, MA, USA). Membranes were then probed with corresponding antibodies against E-cadherin, N-cadherin and β-actin overnight at 4°C, respectively. Subsequently, the protein bands were washed with Tris buffered saline with Tween (TBST) buffer and incubated with horseradish peroxidase (HRP)-conjugated IgG antibody at room temperature (25˚C). Ultimately, the protein bands were visualized using ECL (Electrochemi-luminescence) Substrate and exposed to X-ray film.

### Plasmid transfection

Firstly, LKB1 cDNA was cloned into pcDNA3.1 vector. When laryngeal carcinoma Hep-2 or TU2121 cells reached 80 % confluency for plasmid transfection, cells were transiently transfected with X-treme GENE HP DNA Transfection Reagent (Roche, Germany) for 48 h following the manufacturer's instructions, and prepared for the subsequent experiments.

### Xenograft tumor model

Twelve four-week-old male BALB/C nude mice were obtained from and maintained in the Animal Experimental Center of Xi'an Jiaotong University, Xi'an, China. All the animal experiments were approved by the Ethics Committee of Animal care and Use of Xi'an Jiaotong University. The mice were randomly divided into two groups equally (Control group and oridonin group), and 100 μL serum-free mixture containing 5×10^6^ Hep-2 cells was subcutaneously injected into the right flank of every nude mouse. Then the two different groups were managed with or without oridonin (10 mg/kg). Whereafter, the tumor diameter and body weight of nude mice were evaluated every 3 days, and the tumor volume was calculated by 1/2 × (length) × (width)^2^. Finally, all the nude mice were sacrificed at day 18 and xenografts isolated from mice were prepared for the subsequent western blotting assay. The data from statistical analysis was represented as mean ± standard deviation, and statistical significance (defined as *P* < 0.05) was analyzed by the two-sample t-test of software GraphPad. Prism 6.

## Results

### Oridonin inhibited the proliferation of laryngeal carcinoma cells

**Figure 1:**
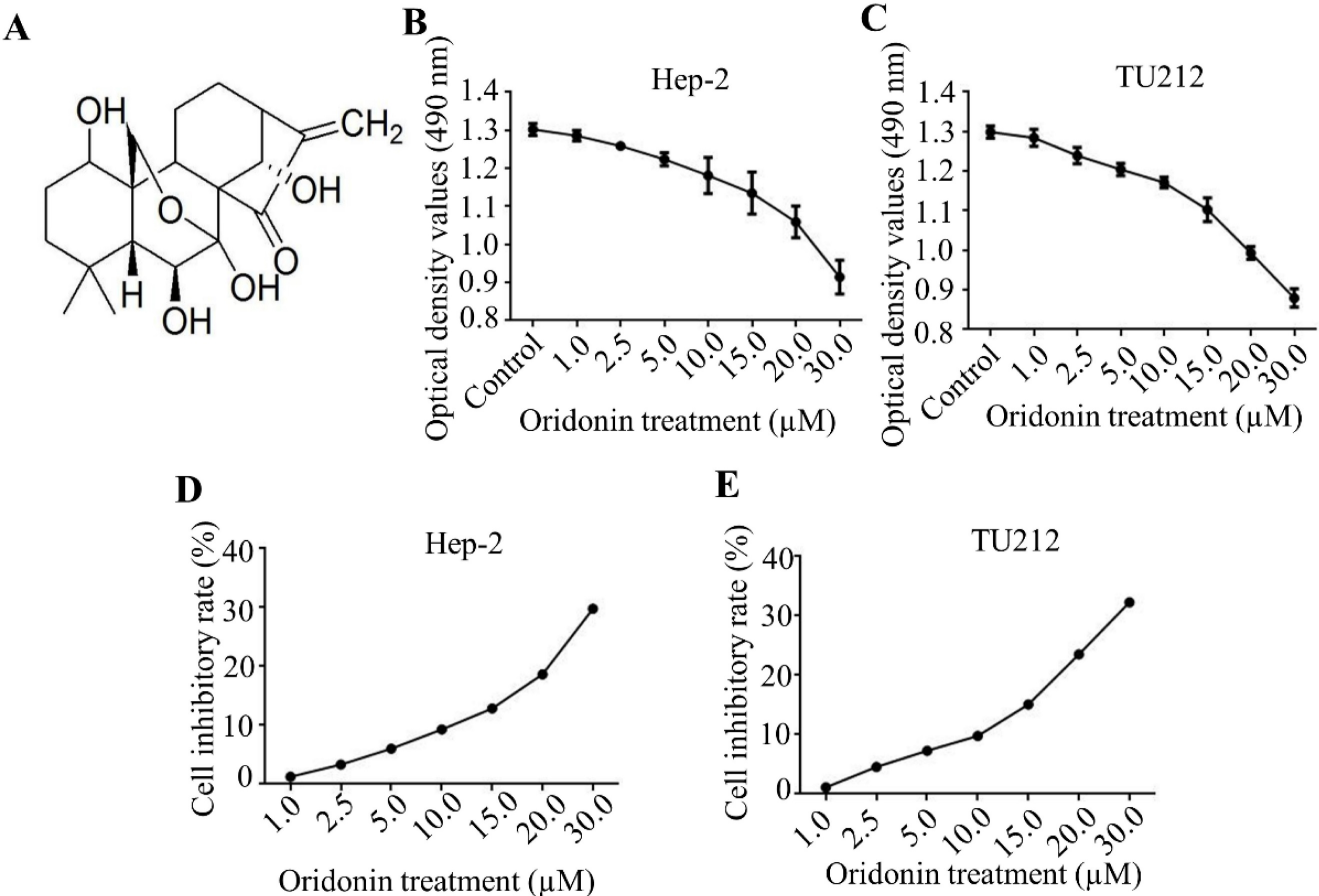
** Oridonin suppressed the proliferation of laryngeal carcinoma cells. (A)** The chemical structure of oridonin. Hep-2 **(B, D)** and TU212 **(C, E)** cells with 90 percent density were exposed to different concentrations of oridonin for 24h (0, 1, 2.5, 5.0, 10.0, 15.0, 20.0, 30.0 μM). Then the viability of laryngeal carcinoma Hep-2 and TU212 cell lines was evaluated by MTT assay. The optical density and cell inhibitory rate were presented. Error bars indicated mean ± SD. Five independent experiments were performed.

Firstly, the molecular structure of oridonin was presented in Fig. 1A. In the present study, the cytotoxic effects of oridonin on laryngeal carcinoma Hep-2 (Figure 1B, 1D) and TU212 (Figure 1C, 1E) cell lines were preliminarily detected by modified MTT assay. The findings uncovered that oridonin had a dose-dependent anti-proliferative effect on laryngeal cancer Hep-2 and TU212 cells (Figure 1B-1E). Meanwhile, under the condition of low concentration of oridonin (no higher than 10 μM), the cytotoxic effect of oridonin on these two laryngeal cancer cells was not significant at 24 h, and the inhibition ratio was less than 10%. Therefore, oridonin with a concentration of 10 μM (0, 2.5, 5.0, 10.0 μM) for 24 h was used for subsequent experiment to eliminate the interference on cell proliferation.

### Oridonin inhibited the metastatic phenotype of laryngeal carcinoma cells

**Figure 2:**
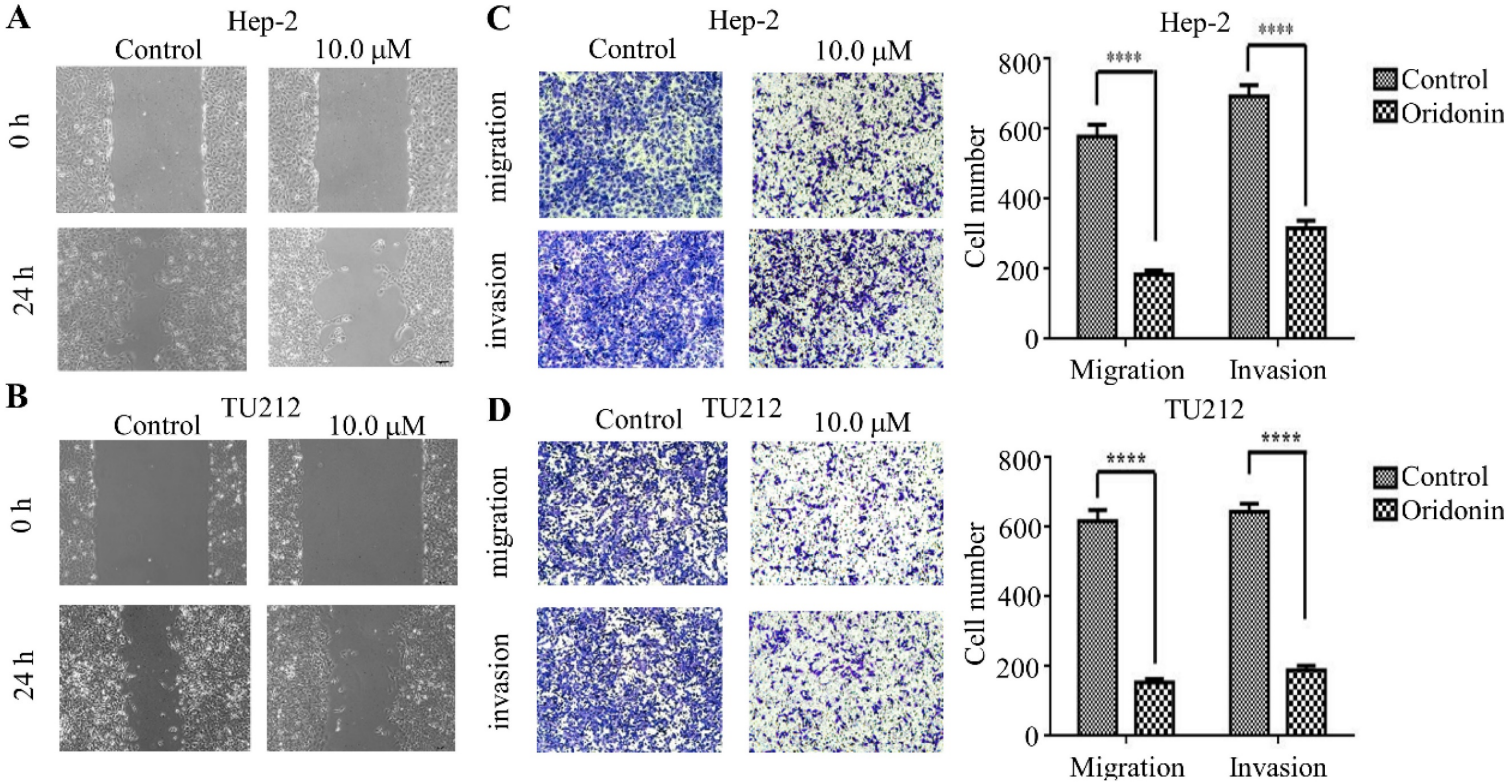
Oridonin repressed metastatic phenotype of laryngeal carcinoma Hep-2 and TU212 cells. Wound healing assay was used for the detection of anti-migratory activity of oridonin on Hep-2 and TU212 cells. The width of scratches was assessed in negative control or oridonin (10 μM) group in Hep2 and TUT212 cells (2A, 2B). Using Transwell migration assay and Matrigel invasion assay, Hep-2 and TU212 cells were exposed to oridonin treatment (10 μM) for 24 h and the number of migrated or invaded cells per chamber was assessed. The experiments were performed in triplicate (****, P<0,0001).

To verify the anti-metastatic effect of oridonin on laryngeal carcinoma cells, the data from wound healing experiment exhibited that the scratch width of oridonin group was much wider than that of the control group in laryngeal carcinoma Hep-2 and TU212 cell lines (Fig.2A and 2B), suggesting an inhibitory role of oridonin in the migration of laryngeal carcinoma cells. Subsequently, the anti-migratory and anti-invasive effect of oridonin were evaluated by transwell migration assay and matrigel invasion assay. As expected, the results exhibited (Fig. 2) that the migratory and invasive ability of Hep-2 and TU212 cell lines was weakened upon oridonin treatment. These data strongly indicated that oridonin had a prominent anti-metastatic effect on laryngeal carcinoma.

### Oridonin regulated expressions of epithelial-mesenchymal transition-related indicators in laryngeal carcinoma cells

**Figure 3:**
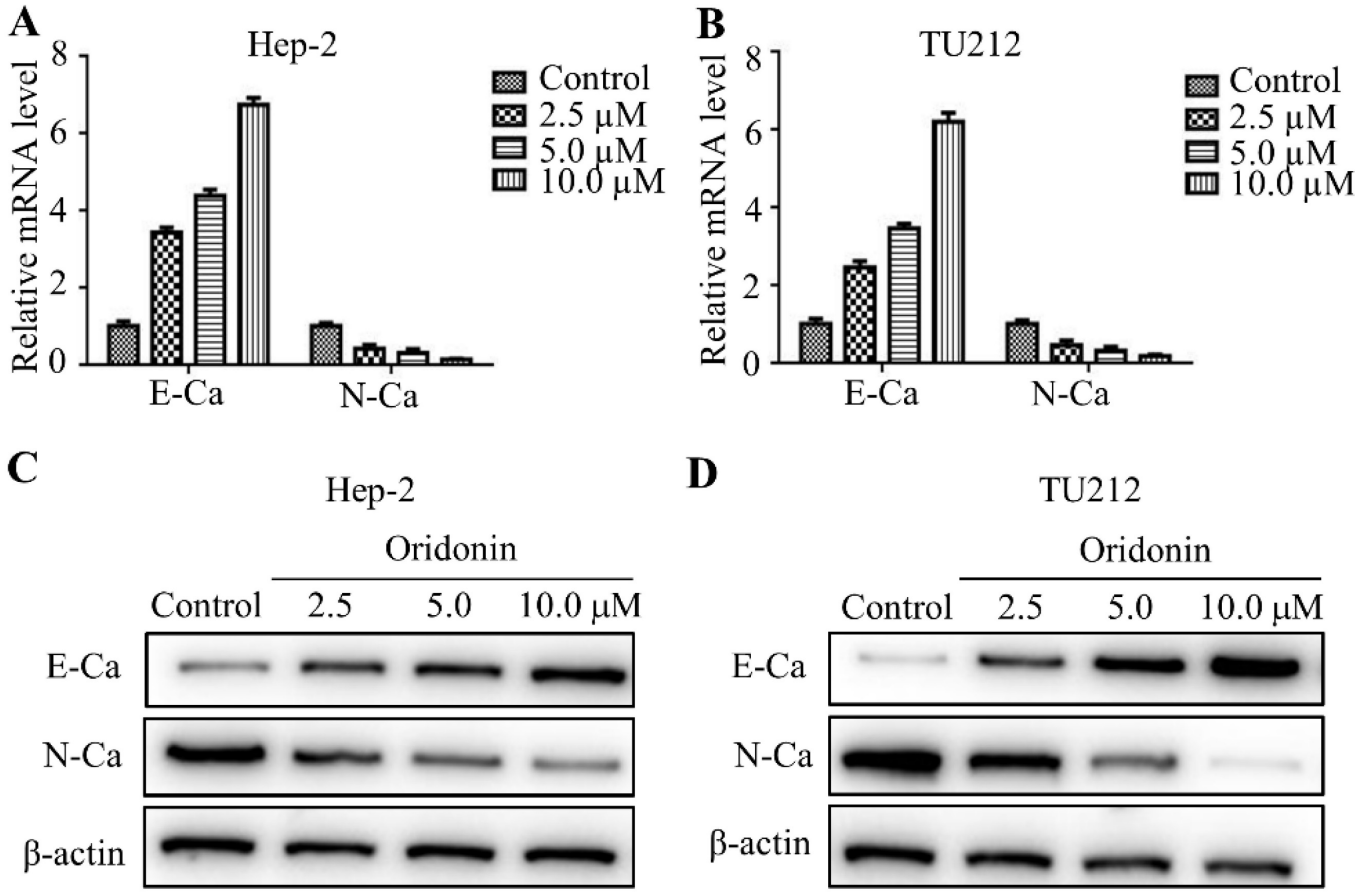
Oridonin remarkably reversed EMT in laryngeal carcinoma cells. Quantitative real-time PCR was used to explore the mRNA level of E-cadherin and N-cadherin in Hep-2 and TU212 cell lines upon oridonin treatment (2.5, 5.0 and 10 μM) **(A and B)**. Hep-2 and TU212 cells pre-treated with certain doses of oridonin (2.5, 5.0 and 10 μM) were subjected to western blotting for E-cadherin (E-Ca), N-cadherin (N-Ca) and β-actin **(C and D)**. Representative protein bands from five experiments were shown. EMT, epithelial-mesenchymal transition.

It has been widely reported that epithelial-mesenchymal transition (EMT) is closely related to tumor malignant progression. Hence in the present study, real-time fluorescence quantitative PCR was used to detect the expression of E-cadherin and N-cadherin in mRNA level. The findings exhibited that oridonin can gradually upregulate the mRNA level of E-cadherin, while downregulate N-cadherin level in a concentration-dependent manner (Fig. 3A and 3B). Meanwhile, oridonin dose-dependently reduced N-cadherin protein level while raised E-cadherin expression (Fig. 3C and 3D). These results revealed that oridonin could reverse epithelial-mesenchymal transition of laryngeal carcinoma cell to some extent.

### Oridonin blocked epithelial-mesenchymal transition of laryngeal carcinoma by positively regulating LKB1/AMPK signaling

**Figure 4:**
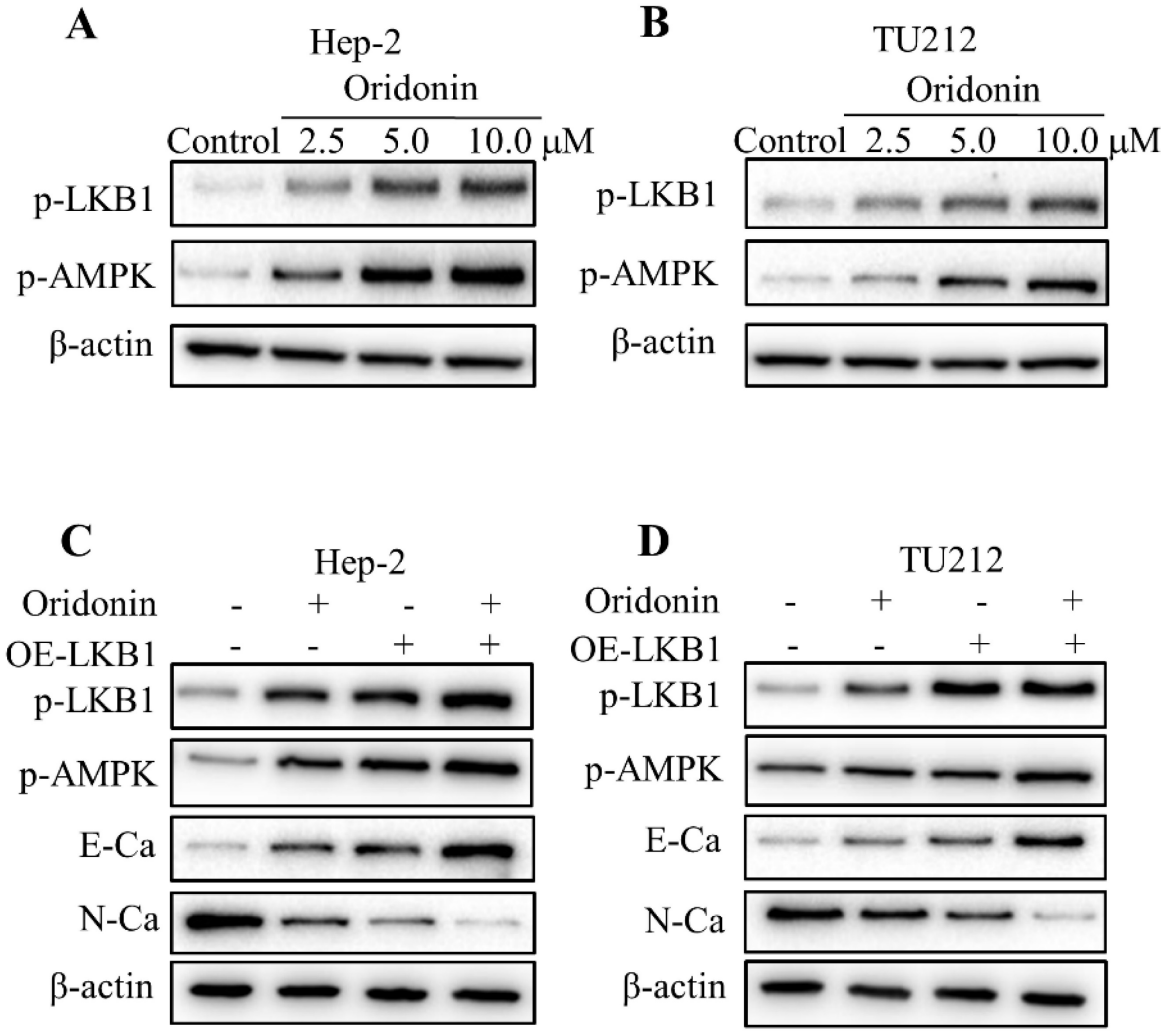
Oridonin reversed EMT of laryngeal carcinoma by upregulating LKB1/AMPK signaling. Hep-2 and TU212 cells pre-treated with certain doses of oridonin (2.5, 5.0 and 10 μM) were subjected to western blotting for phosphorylated-LKB1, phosphorylated-AMPK and β-actin. Representative protein bands from five experiments were shown **(A and B)**. **(C and D)** Cells overexpressing LKB1 by plasmid transfection were synergistically treated with oridonin to detect the change of EMT markers in Hep-2 and TU212 cells. Western blotting was used to analyze the expressions of phosphorylated-LKB1, phosphorylated-AMPK, E-cadherin (E-Ca), N-cadherin (N-Ca) and β-actin. EMT, epithelial-mesenchymal transition.

To validate whether LKB1/AMPK signaling is participated in oridonin's anticancer characteristic, we firstly detected the expressions of LKB1 and AMPK upon oridonin treatment by western blot. As shown in Figure 4A and 4B, phosphorylation levels of LKB1 and AMPK were drastically elevated under certain concentrations of oridonin treatment in Hep-2 and TU212 cell lines. To further verify the role of LKB1 in the inhibition of oridonin on Hep-2 and TU212 cell lines, we transfected LKB1 overexpression with a plasmid. The results presented that overexpression of LKB1 further reinforced the expression of E-cadherin while weakened N-cadherin protein level (Fig. 4C and 4D). Moreover, the downstream kinase of LKB1, p-AMPK, was also up-regulated under LKB1 overexpression. All these results explicitly confirmed that oridonin could inhibit EMT of laryngeal carcinoma via the activation of LKB1.

**Figure 5:**
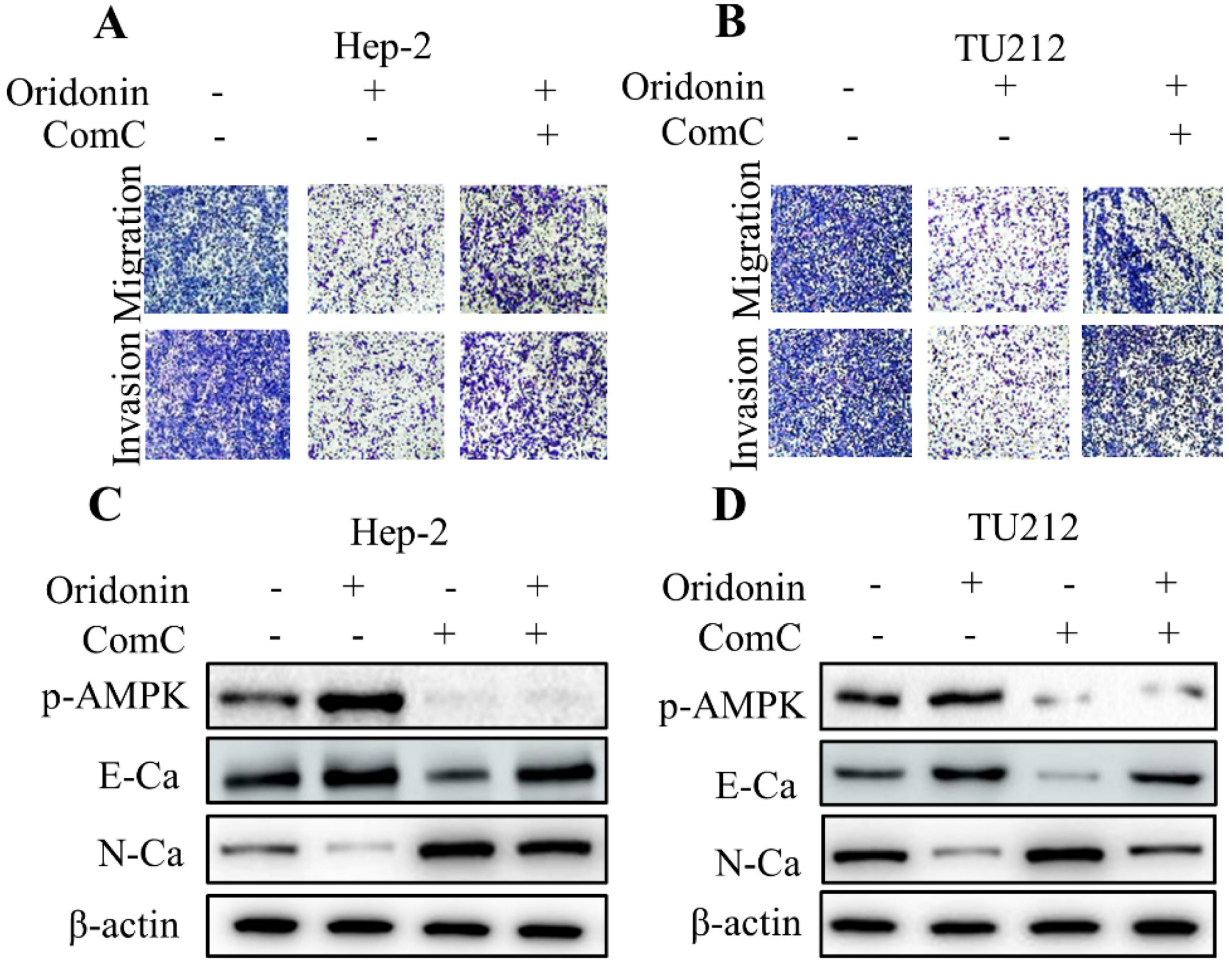
Inactivation of AMPK by Compound C (ComC) attenuated the anti-metastatic effect of oridonin on laryngeal carcinoma cells. **(A and B)** Transwell migration assay and Matrigel invasion assay were used to explore the metastatic phenotype of Hep-2 and TU212 cells with different treatments (oridonin with or without compound C). Five random fields were observed by microscopy. All the experiments were performed in triplicate. **(C and D)** Western blotting was performed to explore the expressions of phosphorylated-AMPK, E-cadherin (E-Ca), N-cadherin (N-Ca) and β-actin in Hep-2 and TU212 cells with different treatment (oridonin with or without compound C). Representative results from five independent experiments were shown.

**Figure 6:**
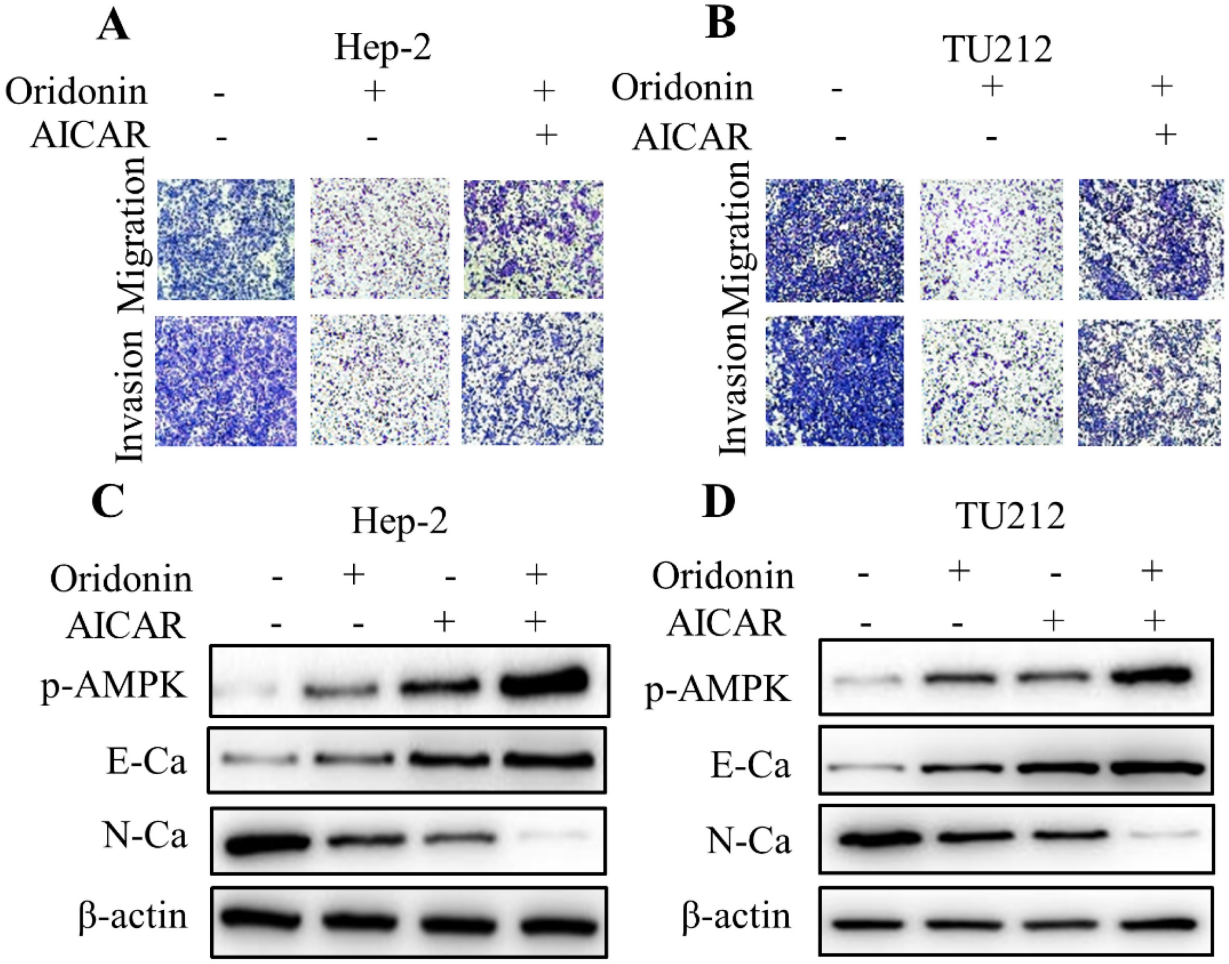
Activation of AMPK by AICAR further weakened migration and invasion, and reversed EMT in oridonin-treated laryngeal carcinoma cells. **(A and B)** Using Transwell migration assay and Matrigel invasion assay, the migrated and invaded Hep-2 and TU212 cells were assessed upon different treatments (oridonin with or without AICAR). (C and D) Hep-2 and TU212 cells co-treated with oridonin and AICAR were immunoblotted for phosphorylated-AMPK, E-cadherin (E-Ca), N-cadherin (N-Ca) and β-actin. Representative results from five independent experiments were shown. EMT, epithelial-mesenchymal transition.

To further shed light on the crucial role of LKB1/AMPK signaling in anti-metastatic effect of oridonin on laryngeal carcinoma, compound C (ComC, AMPK inhibitor) and AICAR (AMPK activator) were used to collaborate with oridonin for the subsequent experiments. The data from transwell migration assay and Matrigel invasion assay uncovered that compound C could partially antagonise the anti-metastatic effect of oridonin on Hep-2 and TU212 cells (Fig. 5A and 5B). Furthermore, Oridonin-mediated upregulation of E-cadherin and downregulation of N-cadherin were attenuated by ComC to some extent (Fig. 5C and 5D). In contrast, the anti-migratory and anti-invasive effects of oridonin on laryngeal cancer Hep-2 and TU212 cell lines were further enhanced by AICAR (Fig. 6A and B). Meanwhile, E-cadherin protein level in Hep-2 and TU212 cells was strengthened under co-treatment with oridonin and AICAR, while the protein level of N-cadherin was declined (Fig. 6C and D). The above data fully confirmed the anti-metastatic characteristic of oridonin by activating AMPK phosphorylation. Taken together, these results revealed that LKB1/AMPK signaling pathway is involved in oridonin-regulated laryngeal carcinoma migration, invasion, and EMT.

### Oridonin inhibited tumorigenicity in vivo

**Figure 7:**
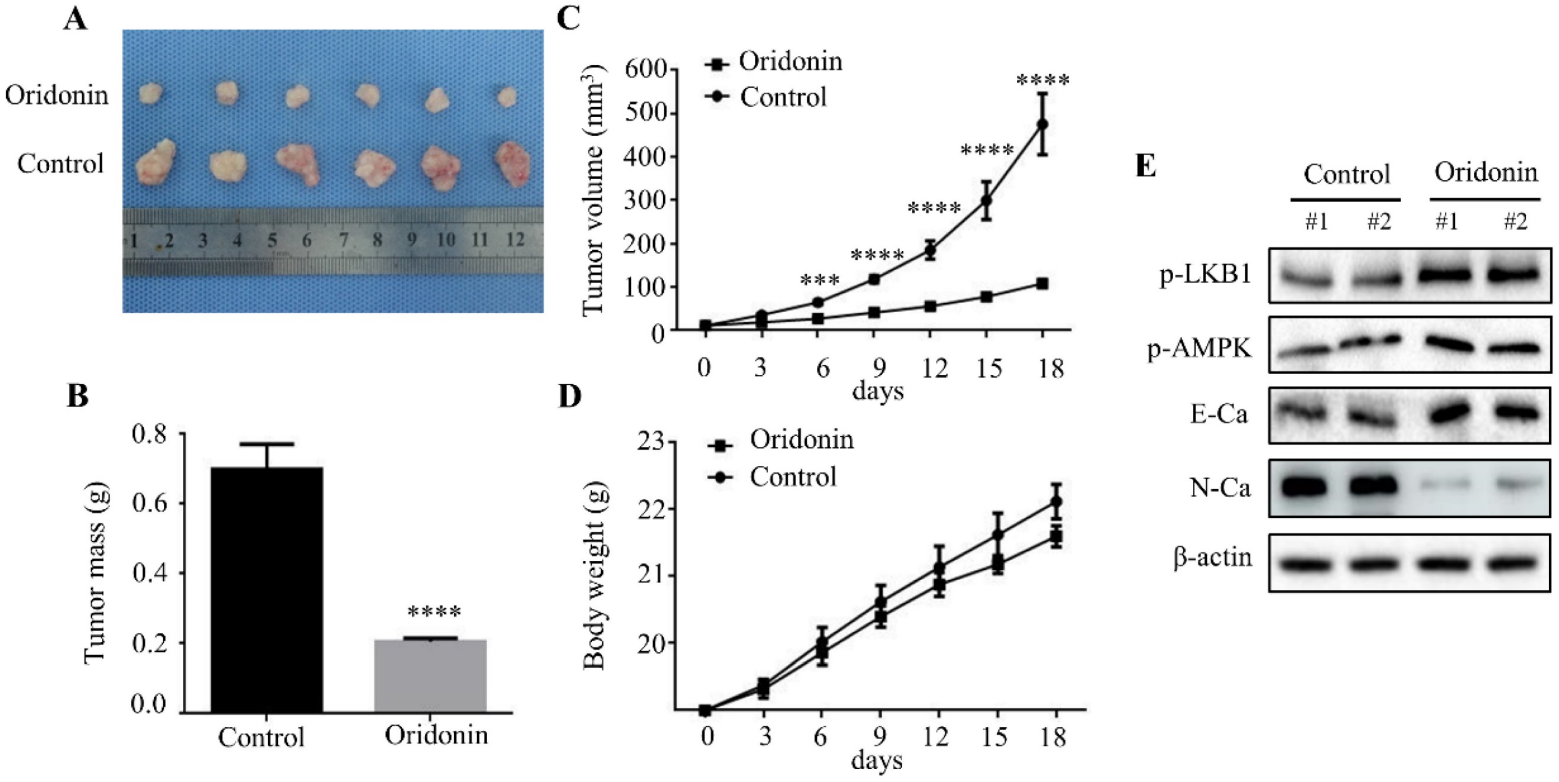
Oridonin prominently decreased laryngeal carcinoma tumorigenicity in a nude mouse xenograft model. **(A)** Representative picture of xenografts in control and oridonin groups were presented at the 18th day. 5 x10^6^ Hep-2 cells were subcutaneously injected into the right flank of nude mice. **(B)** The quantification analysis of tumor mass in control and oridonin groups were shown as mean ± SD (****P<0.0001). **(C)** Tumor volumes and **(D)** body weight of nude mice were detected every 3 days. The statistical results were presented as mean ± SD of five mice (***P<0.001, ****P<0.0001). **(E)** The protein levels of phosphorylated LKB1, phosphorylated AMPK, E-cadherin, N-cadherin and β-actin were detected by Western blot. The proteins were collected from the dissected tumor tissues of control and oridonin groups).

To further clarify the therapeutic effect of oridonin on laryngeal cancer *in vivo*, we established a xenograft tumor model of nude mice using Hep-2 cells. The results exhibited a significant reduction in tumor mass and volume in oridonin group compared to the control group (Fig. 7A-7C), whereas the weight of the two groups was similar (Fig.7D). Subsequently, the data of western blotting showed the ascending level of phosphorylated-LKB1, phosphorylated-AMPK and E-cadherin proteins, and descending level of N-cadherin proteins in tumor tissues exposed to oridonin (Fig. 7E). All the results strongly hinted that oridonin could inhibit laryngeal carcinoma tumorigenesis *in vivo*.

## Discussion

In recent years, attention has shifted to the development of natural anti-tumor drugs 21. Oridonin, isolated from Rabdosia rubescens, is a kind of bioactive diterpenoid compound. In view of its robust anticancer activity in a variety of tumors, nowadays it has been the focus of research 39, 42, 43. Studies reported that oridonin could inhibit metastasis of ovarian cancer by inhibiting mTOR pathway 44. And oridonin was found to negatively regulate Wnt/β-catenin signaling pathway, and to inhibit migration and EMT of pancreatic cancer cells 45. In small cell lung cancer, oridonin may up-regulate the levels of E-cadherin and ALP (Alkaline phosphatase), decrease the levels of vimentin, phosphor-FAK, snail, slug and LDH (Lactate dehydrogenase), and inhibit EMT 46. In terms of anti-breast cancer, oridonin blocked Notch signaling pathway to inhibit cell growth and metastasis, induce autophagy and apoptosis 20. Several studies reported the relationship between oridonin and apoptosis induction in laryngeal carcinoma 24-25, 27-28. However, no studies have explored the role of oridonin in laryngeal carcinoma metastasis. In the present study, it is the first time elucidated that at low concentrations (less than 10 μM), oridonin exhibited a remarkable inhibitory effect on migration and invasion of laryngeal cancer Hep-2 and TU212 cells.

EMT (epithelial-mesenchymal transition) is a complicated biological process of epithelial cells transforming into mesenchymal cells through a specific procedure, which is manifested by loss of inter-cell cohesion, increase of extracellular matrix components, and accelerated cell migration and invasion 47. There is increasing evidence demonstrating that EMT occurs in the development and metastasis of various cancers 47. Hence further research to identify the molecular target of EMT in laryngeal cancer cells may provide a new method for the treatment of laryngeal cancer 48. In our study, the findings from quantification real-time PCR and western blotting presented a prominent inhibitory effect of oridonin on EMT of laryngeal carcinoma, which was in accordance with the previous study.

To verify the underlying mechanism of oridonin on metastatic phenotype and EMT of laryngeal carcinoma, a variety of pathways were under investigation. Previous studies uncovered that LKB1/AMPK signaling pathway was participated in tumor migration and invasion 49. When LKB1 was overexpressed in lung cancer cells, the growth and proliferation of lung cancer cells were declined, while in lung adenocarcinoma cells with LKB1 deletion, ZEB1 expression was elevated 50. It has also been reported that LKB1 overexpression could impair the growth and metastasis of gastric cancer cells 51, 52. In addition, AMPK, the downstream of LKB1, was also closely related to tumor growth 53. It has been found that α-enolase accelerated metastasis of colorectal cancer by negatively regulating AMPK signaling pathway 54. Hence, we speculated whether LKB1/AMPK signaling pathway was involved in the anti-migratory and anti-invasive effect of oridonin on laryngeal carcinoma cells. The data from western blotting proved that oridonin dramatically enhanced the phosphorylation levels of LKB1 and AMPK proteins in Hep-2 and TU212 cell lines. Subsequent studies validated that LKB1 overexpression could strengthen the inhibition of EMT mediated by oridonin, suggesting a vital role of LKB1 in anti-metastatic effect of oridonin on laryngeal carcinoma. Similar with the above results, pre-treatment with AMPK activator AICAR also enhanced oridonin-mediated anti-migratory and anti-invasive effect. In contrast, AMPK inhibitor compound C achieved an opposite effect. The data strongly suggested that oridonin partially reversed EMT of laryngeal carcinoma *in vitro* via the activation of LKB1/AMPK signaling. Consistent with the above results, the *in vivo* experiments further revealed that oridonin obviously restrained tumorigenicity of laryngeal cancer xenografts, which displayed a robust anticancer characteristic of oridonin on laryngeal carcinoma* in vivo*.

In summary, this study validated that oridonin could repress the metastatic phenotype of laryngeal carcinoma and reverse EMT *in vitro* and *vivo* by positively regulating LKB1/AMPK signaling pathway. These results strongly indicated that oridonin may be an optional treatment for laryngeal carcinoma. Additionally, LKB1/AMPK signaling may become a potential therapeutic target for laryngeal carcinoma.
